# An efficient pseudomedian filter for tiling microrrays

**DOI:** 10.1186/1471-2105-8-186

**Published:** 2007-06-07

**Authors:** Thomas E Royce, Nicholas J Carriero, Mark B Gerstein

**Affiliations:** 1 Interdepartmental Program in Computational Biology and Bioinformatics, Yale University, New Haven, CT 06520, USA; 2 Department of Computer Science, Yale University, New Haven, CT 06520, USA; 3 Department of Molecular Biophysics and Biochemistry, Yale University, New Haven, CT 06520, USA

## Abstract

**Background:**

Tiling microarrays are becoming an essential technology in the functional genomics toolbox. They have been applied to the tasks of novel transcript identification, elucidation of transcription factor binding sites, detection of methylated DNA and several other applications in several model organisms. These experiments are being conducted at increasingly finer resolutions as the microarray technology enjoys increasingly greater feature densities. The increased densities naturally lead to increased data analysis requirements. Specifically, the most widely employed algorithm for tiling array analysis involves smoothing observed signals by computing pseudomedians within sliding windows, a *O*(*n*^2^log*n*) calculation in each window. This poor time complexity is an issue for tiling array analysis and could prove to be a real bottleneck as tiling microarray experiments become grander in scope and finer in resolution.

**Results:**

We therefore implemented Monahan's HLQEST algorithm that reduces the runtime complexity for computing the pseudomedian of *n *numbers to *O*(*n*log*n*) from *O*(*n*^2^log*n*). For a representative tiling microarray dataset, this modification reduced the smoothing procedure's runtime by nearly 90%. We then leveraged the fact that elements within sliding windows remain largely unchanged in overlapping windows (as one slides across genomic space) to further reduce computation by an additional 43%. This was achieved by the application of skip lists to maintaining a sorted list of values from window to window. This sorted list could be maintained with simple *O*(log *n*) inserts and deletes. We illustrate the favorable scaling properties of our algorithms with both time complexity analysis and benchmarking on synthetic datasets.

**Conclusion:**

Tiling microarray analyses that rely upon a sliding window pseudomedian calculation can require many hours of computation. We have eased this requirement significantly by implementing efficient algorithms that scale well with genomic feature density. This result not only speeds the current standard analyses, but also makes possible ones where many iterations of the filter may be required, such as might be required in a bootstrap or parameter estimation setting.  Source code and executables are available at .

## Background

**Figure 1:**
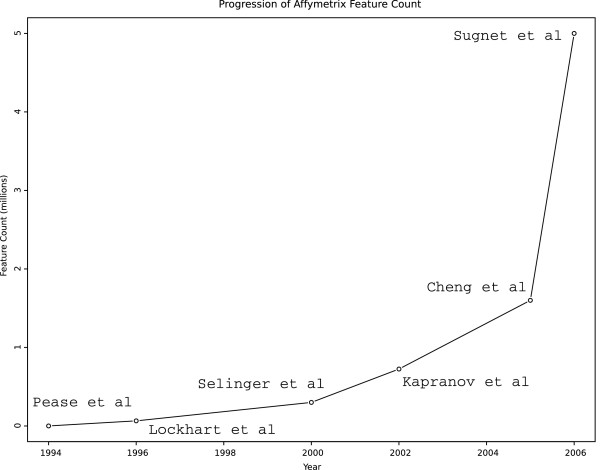
**Number of features per Affymetrix brand microarray**. Example studies' [1; 13–17] feature count are plotted as a function of their publication year.

A descendant of DNA microarray technology, the tiling microarray allows unbiased, high resolution interrogation of genome function [1]. Generally, such investigations consist of querying labeled nucleic acid samples with tethered DNA probes that target regularly spaced tiles from a known genomic sequence [2]. These tiles' average spacing can be decreased and/or their genomic coverage can be increased with improvements in microarray feature density, which certainly appears to be occurring (Figure 1). Effective feature density can be further increased by synthesizing multiple probes within a single feature in so-called 'double-tiled' array designs [3]. Such high feature densities allow large-scale functional genomics capabilities that have made tiling microarrays one of the ENCODE consortium's major enabling technologies [4].

Given their utility and increasing resolution, tiling microarray data processing algorithms that scale well with advances in feature density and are available in usable software are generally desirable. A hurdle to the efficiency goal is that, like their gene-centric counterparts, tiling microarrays generate data that are most successfully analyzed with statistically robust techniques that aggregate signal measured from multiple different features targeting the same nucleic acid [5]. This adds a level of complexity to the efficient design of analysis algorithms since statistically robust procedures are typically more complicated than their less-robust counterparts.

The goal of most said analysis algorithms is to identify those regions of the tiled genomic sequence that yield higher than expected signal intensities. A variety of approaches have been suggested for this task, including simple intensity thresholding [6], correlation of genomic neighbor features' array signals [7], proximity-based heuristics [8,9], dynamic programming [10], and hidden Markov models [11-14]. Factor graphs have been successfully applied to analyzing exon tiling array data [15] and there appears to be no restraints on their application to genomic tiling arrays. The different strategies listed here each have their own advantages. Intensity thresholding and the various proximity-based heuristics are conceptually simple – the brightest spots on the array should correspond to active regions, and stretches of such features along a chromosome provide additional evidence. Correlation based methods, which include factor graph approaches, have the advantage of using information from many hybridizations and avoid some of the common pitfalls of array analysis like background hybridization [16]. Factor graphs have the additional advantage of easily being able to incorporate other forms of genomic information to aid in the segmentation. Finally, factor graphs along with hidden Markov models and dynamic programming are attractive in that they have their footing in rigorous mathematics.

For several of the approaches listed above (particularly the heuristics), pre-segmentation smoothing can be beneficial. In fact, this processing step is the most widely applied technique in analyzing tiling microarray data and involves replacing every feature's measured signal with a robust, smoothed value thereof. The value typically employed is the pseudomedian of signals reported by features within a fixed genomic distance from the feature (a sliding window) being smoothed [9]. The pseudomedian is often defined as the median of all *n*(*n*+1)/2 pairwise averages among a list of *n *observations and is a robust estimator of central tendency [17]. An equivalent definition of the pseudomedian is the value *μ *that satisfies

0 = ∑ rank (|*X*_*i *_- *μ*|) × sign (*X*_*i *_- *μ*)

where *X*_*i *_represents the *i*th value in a collection of observations. When interpreted this way, *μ *is a root of the one-sample Wilcoxon test statistic [18], or put more simply, a value from which the observations do not significantly deviate.

If one chooses to calculate the pseudomedian directly from its definition as the median of all observations' pairwise averages, this forces a *O*(*n*^2^log*n*) calculation. To arrive at this time complexity, we note that there are *O*(*n*^2^) pairwise averages that need to be sorted to find their median and that this sort dominates the computation. This gives us *O*(*n*^2^log(*n*^2^)) = *O*(*n*^2^(2log*n*)) = *O*(*n*^2^log*n*). Currently, this is the method implied in the tiling microarray literature for sliding window pseudomedian smoothing [9]. As feature densities increase (Figure 1), more features are typically contained within the sliding window. This of course leads to super-quadratically longer computation within each window and can be a burden to large-scale projects employing the tiling microarray technology. In many tiling microarray analyses, the computation of pseudomedians is the only analysis module that scales super-linearly with feature counts per window and therefore dominates overall runtimes, in practice.

To reduce the burden of pseudomedian computations, especially as feature densities increase, we first deployed a *O*(*n*log*n*) solution for computing the pseudomedian. This improvement greatly reduced the theoretical runtime of this calculation and we found that, as to be expected, this solution significantly improved runtimes of tiling microarray smoothing. We next observed that this solution's theoretical properties carry with it a sizable constant factor. We therefore investigated an adjustment to the solution utilizing skip lists that greatly reduces this constant factor and, in practice, cut run times of the pseudomedian filter by a further forty-three percent for a representative test case. Both algorithms are significant improvements over the definition-derived implementation of the sliding window pseudomedian filter commonly used in tiling microarray analysis.

## Results and discussion

### Algorithm

#### Implementation of a pseudomedian filter from its definition

**Figure 2:**
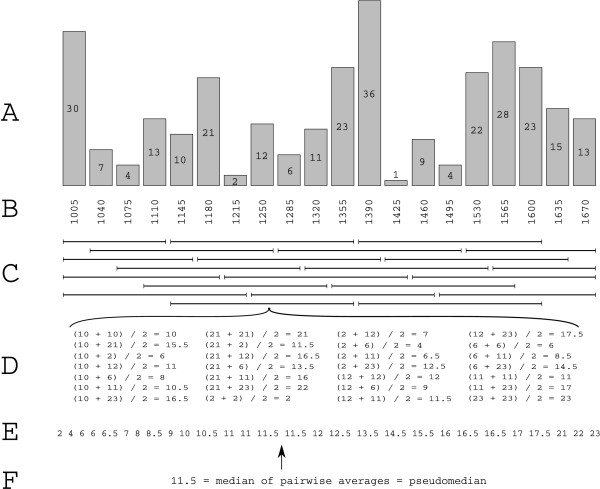
**Computation of sliding window pseudomedianutilizing a 115 bp bandwidth**. Raw signals are depicted in (A) along with their genomic coordinates (B). Pseudomedians are computed in a sliding window (C). As an example, (D) tabulates all 28 pairwise average in the window centered at position 1,250. The averages are sorted in (E) from which their median can be computed (F). The median of pairwise averages is also called the pseudomedian.

Inputs to any pseudomedian filtering algorithm consist of a vector of ordered genomic coordinates and corresponding feature intensities acquired from a tiling microarray hybridization. The goal is to replace the observed intensity of feature *f *with the pseudomedian of those features' intensities that lie within a span of *b *nucleotides from *f*'s genomic position (Figure 2).

If there are *n *probes within *b *nucleotides of *f*, then computing the pseudomedian estimate requires first computing all *n*(*n*+1)/2 pairwise averages among the *n *features, and then computing the median of these values. This algorithm is computed for each feature in a sliding window across genomic coordinates. A common practice is to combine replicate microarrays' data into this calculation. That is, if there are *m *replicate arrays, each window will contain *nm *features. This modification results in a dramatic increase in runtime in that this implementation will require the computation of *O*((*mn*)^2^) pairwise means within each window. Therefore, so as to avoid any confusion, when we refer to *n *features within a window we will be assuming that this *n *includes any replicate data counts.

#### A more efficient pseudomedian algorithm

To reduce the time complexity of computing pseudomedians, we have implemented the algorithm of Monahan [19]. Prior to describing this algorithm, we will first describe another *O*(*n*^2^log*n*) algorithm for computing a pseudomedian. In doing so, we will set the foundations for Monahan's algorithm.

##### Another *O*(*n*^2^log*n*) option

This algorithm takes as its input a list of values *X*_1_, ... *X*_n _and first computes their *n*(*n*+1)/2 pairwise averages,

*S*_0 _= {(*X*_*i *_+ *X*_*j*_)/2, 1 ≤ *i *≤ *j *≤ *n*}.

**Figure 3:**
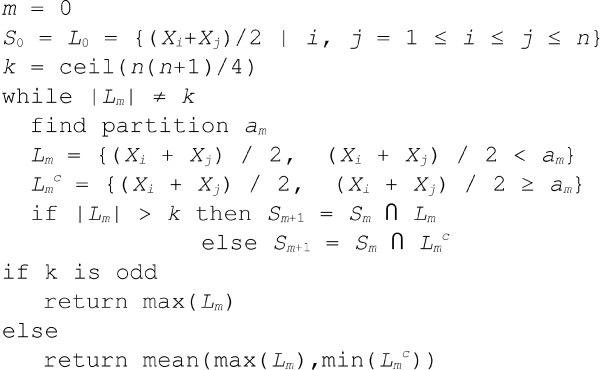
**Pseudocode for the pseudomedian algorithm**. For illustration purposes, this pseudocode assumes no ties in *X*.

These averages are then processed iteratively by (randomly) choosing a partition element *a*_*m *_during the *m*th iteration and splitting *S*_0 _into two sets: one containing those elements of *S*_0 _less than *a*_*m *_and a second containing those elements greater than or equal to *a*_*m*_. The value *a*_*m *_can also be thought of as a *best guess *at the set's pseudomedian which we will iteratively refine. *S*_*m*+1 _is then defined to be the intersection of *S*_*m *_and the larger of the two partitions. Since we always intersect *S*_*m *_with the larger of the two groups, the intersection must always contain the median. (Note that since we're dealing with the pairwise averages of *X*_1_, ... *X*_*n*_, this median value is actually the desired pseudomedian.) This iterative partitioning is continued until *S*_0 _is evenly split (to simplify the presentation, we are assuming here that *S*_0 _has an odd number of elements, no two of which are equal). At this point, the pseudomedian is simply the maximum of those values within *S*_0 _which are less than the final *a*_*m*_. In pseudocode, this algorithm can be written as in Figure 3.

If implemented, the algorithm described above would require *O*(*n*^2^) computations to compute just a single iteration of the partitioning procedure. We can expect that, on average, this procedure is iterated *O*(log(*n*^2^)) = *O*(log*n*) times because *S*_*m *_can be expected to be half as big after each iteration, given randomly chosen values of *a*_*m*_. This results in an overall runtime complexity *O*(*n*^2^log*n*).

##### An *O*(*n*^2^) algorithm

**Figure 4:**
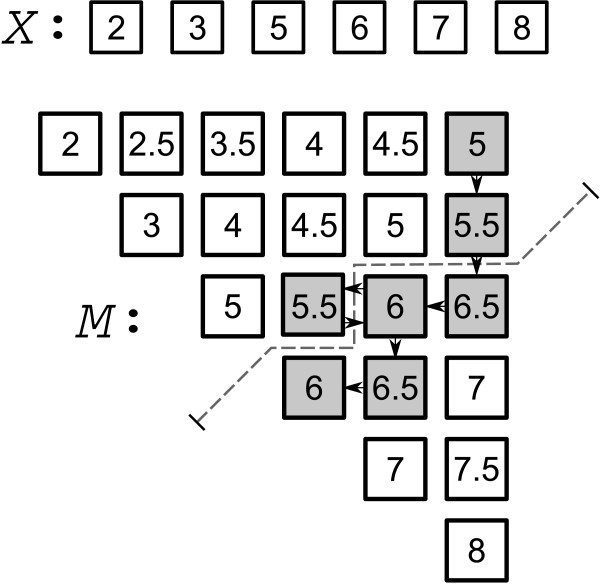
**Partitioning the pairwise means in linear time**. A partition is indicated by a dotted line such that all elements above and to the left of the line are strictly less than six. The pairwise averages that are required to be computed for determining the partition are indicated by the path. Specifically, imagine that the current best guess at the pseudomedian is six – that is, we now want to divide S_0_ into those averages less than six and those greater than or equal to six. We start at the top and right of the matrix and encounter a value of 5. This is less than six so we move down one row. Again, we encounter a value, 5.5, that is less than six so we move down one more row. Note that we now know every element in these previous two rows are less than our partitioning element and that we determined this to be so by computing just two pairwise averages. Returning to the partitioning, we next encounter the value 6.5 which is greater than six, so we scan the row to the left until we find the first element, 5.5, which is less than our partitioning element. When this occurs, we move down a row from six, encounter 6.5, move left, find the value six and reach the diagonal, which completes the partitioning. Importantly, we reached this diagonal by computing only seven pairwise averages. The facilitating requirement of the input data is that it is sorted. This is the partitioning technique implemented in Monahan's algorithm.

An improvement in runtime can be had if the input list *X *is sorted to begin with. Specifically, the partitioning step of the previous algorithm can be found in linear time. We will continue to assume that all pairwise averages are computed upfront, requiring *O*(*n*^2^) computation. With these averages, one can construct an upper-triangular matrix *M *consisting of the elements in *S*_0 _where matrix entry *M*_*i*,*j *_is the average of *X*_*i *_and *X*_*j*_, such that *i *≤ *j *and *X*_*i *_≤ *X*_*j*_. Then, for each row, we have *M*_*i*,*j *_≤ *M*_*i*,*j*+1_, and for each column we have *M*_*i*,*j *_≤ *M*_*i*+1,*j*_. The partitioning of *S*_0 _can be found by traversing *M *top-to-bottom and right-to-left until the diagonal is reached as illustrated in Figure 4. By constructing *M *in this way, the *O*(*n*^2^) elements can be partitioned in the time it takes to reach the diagonal, which is *O*(*n*). We still require *O*(log*n*) partitioning steps, so the pseudomedian can be found in *O*(*n*log*n*) time, assuming that the *O*(*n*^2^) averages have been computed. The initial sorting of *X *requires *O*(*n*log*n*) time so the overall complexity is dominated by computing the pairwise averages. Therefore, the runtime of this algorithm is expected to be *O*(*n*^2^).

##### Monahan's *O*(*n*log*n*) algorithm

**Figure 5:**
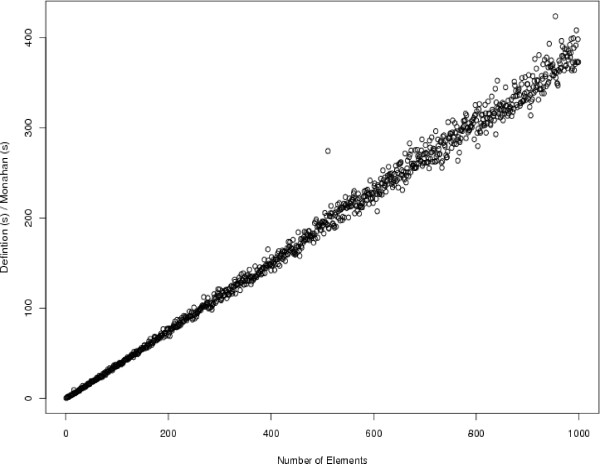
**Observed runtime differences between computing the pseudomedian from its definition and by following Monahan's algorithm**. On the *x*-axis is the number of randomly generated numbers for which the pseudomedian was desired. The *y*-axis is the result of dividing the definition's runtime by Monahan's runtime on the same set of values. The ratio scales linearly with number of elements as predicted from theory. The definition requires a *O*(*n*^2^log*n*) computation whereas Monahan's algorithm needs just *O*(*n*log*n*) computing time; we therefore expect a linear increase in their runtime ratio as *n *increases, and this is what we observe.

The important aspect of the previous matrix partitioning is that it can be done without actually computing all elements of the matrix upfront. Rather, pairwise averages can be computed only when required of the algorithm. This is also illustrated in Figure 4. Since all pairwise averages need not be computed upfront, the runtime analysis follows that of the previously described algorithm, except that the dominating term is now *O*(*n*log*n*) as opposed to *O*(*n*^2^) previously. We compared the in-practice runtimes of computing the pseudomedian with this algorithm with computing the pseudomedian from its definition in Figure 5 to test the theory and found that the runtimes do scale as expected.

As is the case with all 'divide and conquer' styled algorithms, choosing good partitioning elements is crucial for achieving good in-practice running times. Since we are computing pseudomedians in a sliding window for our application, we set the initial partitioning element (or our initial guess at the true pseudomedian), *a*_0_, to be the previous window's pseudomedian. This will approximately split the current window's set of pairwise averages in half. For subsequent values of *a*_*m*_, we pick a row median from *M *at random. Since each row is already 'sorted', this can be done in constant time at each iteration.

#### Maintaining the sliding window

One of the most expensive steps of this pseudomedian algorithm is the initial sorting of elements in *X*. Since from window to window, we expect a relatively small number of elements to be removed and added, we can replace the costly sorting routine with fast insertions and deletions into a sorted list. The sorted list is, of course, just the list used in the previous window's pseudomedian calculation. There are a number of alternative data structures that can maintain a sorted list of numbers. In this work, we chose to implement our sorted list as a skip list [20]. The skip list is easy to implement and insertions and deletions can be performed in log*n *expected time, where *n *is the number of elements in the list.

**Figure 6:**
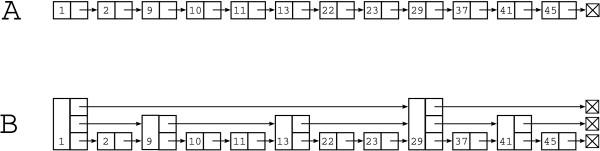
**Linked list (A) and skip list (B)**. The skip list contains nodes with a variable number of forward pointers. A node's level is the same as the number of forward pointers it has. Insertion and deletion into the linked list requires a simple traversal of the list, requiring *O*(*n*) time. Insertion and deletion into/from the skip list can make use of short-cuts provided by the structure. For example, to insert '30' into (B), one would start at the node having value '1', skip ahead to the node holding '9', skip ahead to '13' and then to '29' before encountering '41', which is greater than the value to insert. The traversal would then visit the node having '37' and finally insert between '29' and '37'. Such an operation can be shown to have *O*(*n*log*n*) expected time.

Briefly, a skip list is simply a type of linked list data structure. The main difference between skip lists and linked lists is that each node of the skip list has an associated 'level' attribute. A node's level is determined probabilistically when it is created such that a node of level one is the most likely to occur in the list, level two is the second most likely, and so on. Every node in a skip list contains a datum field analogous to the linked list, but each node has as many forward links as its determined level (Figure 6). For example, a node of level two will have two forward pointers. One of these pointers will point to the next node having level of at least one. The second pointer will point to the next node having level of at least two, and so on.

Utilizing this data structure allows us to omit the sorting step of the modified pseudomedian algorithm and replace it with a few insert and/or delete operations within each window. While big-*O *time complexity of our pseudomedian filter is not improved upon by this modification, we expect at least some benefit in practice.

### Testing

**Figure 7:**
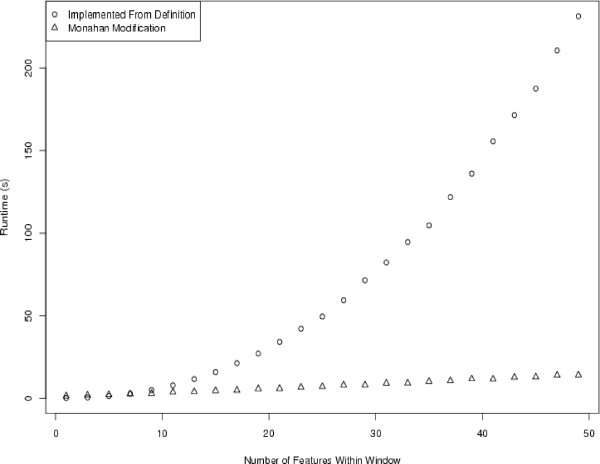
**Runtimes of the pseudomedian smoothing algorithms over a one million element dataset**. Times are plotted for the definition-derived implementation and for the Monahan modification as a function of feature count within the sliding window.

To test the theory, we first implemented the algorithm of Monahan and integrated it with our sliding window code for tiling microarray analysis. We first generated a synthetic dataset of one million features, giving each feature a random intensity and assigning chromosomal positions such that adjacent features were 1 nt apart. This allowed us to easily assess how observed run times scale with the number of features within each sliding window. The definition-derived algorithm's runtimes were compared with the modified algorithm's runtimes for a number of window sizes in Figure 7. Moving beyond synthesized data, we also ran the two different implementations of the pseudomedian filter on ENCODE ChIP-chip data, using the same window span (250 nt) used in the original study. We found that the simpler algorithm ran in 36.76 seconds while the routine using Monahan's pseudomedian algorithm ran in just 3.74 seconds.

**Figure 8:**
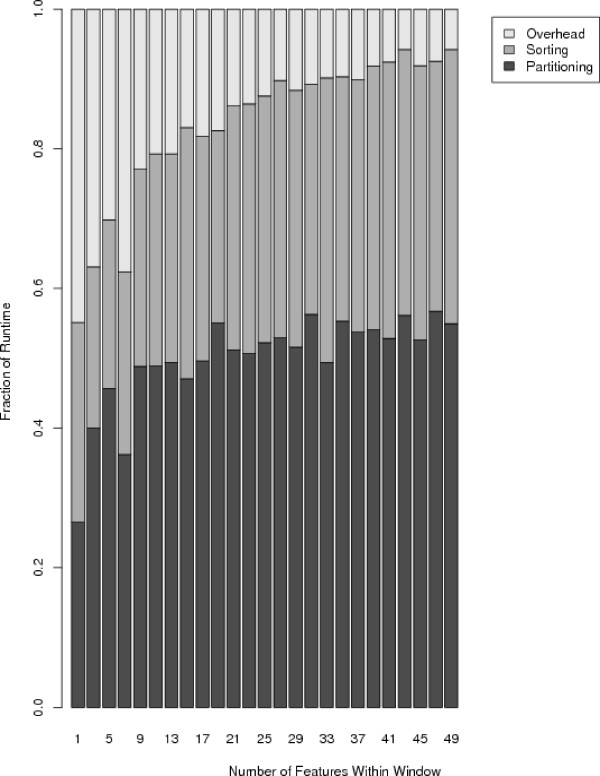
**Fractional runtimes for the Monahan-modified pseudomedian filter**. 'Sorting' refers to time spent sorting values within each window, 'Partitioning' refers to time spent searching for the pseudomedian, and 'Overhead' is the remainder of runtime within the filter.

Next, we hypothesized that the sorting routine required by the Monahan algorithm comprised a large fraction of its overall runtime within each window. To test this hypothesis, we ran our modified algorithm on the same one million data point set with various window spans, recording the amount of computation required for (1) sorting, for (2) the remainder of the pseudomedian routine (partitioning) and for (3) overhead in maintaining sliding windows. The results of this analysis are plotted in Figure 8. We subsequently calculated that the average percent of runtime consumed by sorting was approximately 45%.

**Figure 9:**
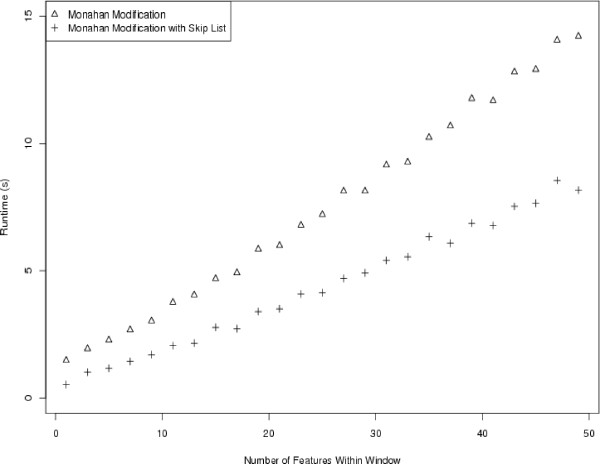
Runtimes for pseudomedian smoothing using the Monahan modification with and without skip list.

To free a bulk of this runtime, we designed our algorithm to maintain a sorted list of intensities from window to window, removing the need to perform a sort within each window. We implemented the sorted list as a skip list [20] and integrated this approach into our code. A comparison of pseudomedian smoothing runtimes between performing sorts in each window and maintaining the sorted list is illustrated in Figure 9. By maintaining the sorted list between windows, runtimes were reduced by approximately 43%, on average in our synthetic dataset and the runtime for our ChIP-chip analysis is reduced to just 2.6 seconds.

### Implementation

The main result of our work is the implementation of these improvements in a downloadable piece of software available at . The program is designed to run at the command-line and we provide precompiled versions for the Linux and Microsoft Windows operating systems. Source code, written in ANSI-C, is available as well for those wishing to run this software on an alternate operating system.

## Conclusion

Post-genome technologies generate vast amounts of data. To arrive at biological inferences, this data must be processed in such a manner that is (1) accurate and (2) efficient. For tiling microarrays, accuracy is best obtained with robust statistical procedures [21]. However, the technique most often used to achieve this robustness is seemingly inefficient in its computation. To relieve this burden, we have suggested the use of Monahan's algorithm for the computation of the pseudomedian. This alteration yields great improvements in runtimes for pseudomedian smoothing. Upon examining this algorithm it became clear that a numeric sort in each window was responsible for much of its clock time. We therefore sought to remove this costly sort and instead decided to maintain a sorted list from window to window since neighboring windows largely consist of the same elements. We implemented a skip list to this end and found this modification increased efficiency by some 43%. There are many data structures that could be used to maintain the sorted list. Various kinds of balanced binary search trees (eg red-black trees) can fill this role, for example. We have implemented the skip list purely out of ease of implementation. The sacrifice we make for this simplicity is that binary search trees can guarantee *O*(log*n*) worst case run times for inserts and deletes while the skip list can offer only *O*(log*n*) *expected *runtimes. Furthermore, inserts and deletes can take quadratic time in the worst case scenario with skip lists. However, our application requires upwards of several million inserts and deletes to/from our skip list, so expected case considerations are realized.

In demonstrating the improved efficiency of our algorithms, we used a synthetic dataset which consisted of one million features, spaced at a constant interval of 1 nt. Doing so simplified the analysis and presentation of runtimes since we could refer to the number of features within a window as opposed to an average genomic spacing as is the most common strategy of tiling microarray construction. Assessing the algorithm in terms of feature counts within windows also allows for the general analysis of increased feature count due to higher tiling density and due to the inclusion of replicate tiling microarrays. To demonstrate practical gains, we have used the algorithms described here to smooth ChIP-chip data generated as part of the ENCODE project. We found that the original pseudomedian filter's runtimes can be improved by 93%.

It is worth noting here that once prohibitive multi-pass analyses become much more feasible with the algorithms we have described. An example multi-pass analysis might aim to place error bars on the estimated pseudomedians. This could be achieved by repeatedly sampling independent hybridizations (with replacement) and performing pseudomedian smoothing on these bootstrap samples. Confidence intervals could then be readily estimated from the samples. Other multi-pass analyses might include finding the optimal span to use in the pseudomedian filter, or in selecting a set of hybridizations that, when smoothed, yield the most satisfying results.

We have not assessed the sensitivity and/or sensitivity gained by using pseudomedian filters in tiling array analyses as these are beyond our scope of computational efficiency. Nonetheless, given the large number of segmentation methods available, the field is probably ripe for such an analysis and we hope to see a comprehensive review soon, building on the recent work of [21]. Hopefully our new algorithms will play a role in such an analysis.

It is our belief that tiling microarray datasets will continue to grow in size and in tiling density. We also expect that their popularity will continue to increase as they are adopted for a growing number of genomic applications. These advances make efficient algorithms such as those presented here and software that implements them of timely need.

## Methods

### Datasets

For analyzing runtimes of the algorithms described here, we primarily rely upon a synthetic set which consists of one million features having intensities drawn from a standard normal curve. To demonstrate utility on a real-world dataset, we downloaded an ENCODE ChIP-chip dataset consisting of 382,884 features and three replicate hybridizations. This array consists of features that represent genomic tiles spaced thirty-eight nucleotides apart. This data is available for download from the Gene Expression Omnibus (GEO) website [22] under series id GSE2714.

## Authors' contributions

TER conceived of the study, wrote and tested code, performed runtime analyses, and drafted the original manuscript. NJC participated in time complexity and runtime analyses as well as drafting the manuscript. MBG contributed to drafting the manuscript and project coordination. All authors read and approved the final manuscript.
